# Dietary *Salicornia ramosissima* improves the European seabass (*Dicentrarchus labrax*) inflammatory response against *Photobacterium damselae piscicida*

**DOI:** 10.3389/fimmu.2024.1342144

**Published:** 2024-03-04

**Authors:** Marina Machado, Francisco Cruz, André Cunha, Lourenço Ramos-Pinto, Adriana Laranjeira, Mário Pacheco, Rui J. M. Rocha, Benjamín Costas

**Affiliations:** ^1^ Interdisciplinary Centre of Marine and Environmental Research (CIIMAR), University of Porto, Matosinhos, Portugal; ^2^ Escola Superior de Turismo e Tecnologia do Mar de Peniche, Instituto Politécnico de Leiria, Peniche, Portugal; ^3^ School of Medicine and Biomedical Sciences (ICBAS-UP), University of Porto, Porto, Portugal; ^4^ Riasearch, Lda., Murtosa, Portugal; ^5^ Centre for Environmental and Marine Studies (CESAM) and Department of Biology, University of Aveiro, Aveiro, Portugal

**Keywords:** aquaculture, sustainability, phagocytes, molecular immunology, nutritional immunology

## Abstract

**Introduction:**

Modern fish farming faces challenges in sourcing feed ingredients, most related with their prices, 21 availability, and specifically for plant protein sources, competition for the limited cultivation space for 22 vegetable crops. In that sense, halophytes have the added value of being rich in valuable bioactive compounds and salt tolerant. This study assessed the inclusion of non-food fractions of *S. ramosissima* in European seabass diets.

**Methods:**

Different levels (2.5%, 5%, and 10%) were incorporated into seabass diets, replacing wheat meal (diets ST2.5, ST5, and ST10) or without inclusion (CTRL). Experimental diets were administered to seabass juveniles (8.62 ± 0.63 g) for 34 and 62 days and subsequent inflammatory responses to a heat-inactivated *Photobacterium damselae* subsp. *piscicida* (*Phdp*) were evaluated in a time-course manner (4, 24, 48, and 72 h after the challenge). At each sampling point, seabass haematological profile, plasma immune parameters, and head-kidney immune-related gene expression were evaluated.

**Results:**

After both feeding periods, most parameters remained unaltered by *S. ramosissima* inclusion; nonetheless, seabass fed ST10 showed an upregulation of macrophage colony-stimulating factor 1 receptor 1 (*mcsf1r1)* and cluster of differentiation 8 (*cd8β)* compared with those fed CTRL after 62 days of feeding. Regarding the inflammatory response, seabass fed ST10 showed lower plasma lysozyme levels than their counterparts fed ST2.5 and ST5 at 24 h following injection, while 4 h after the inflammatory stimulus, seabass fed ST10 presented higher numbers of peritoneal leucocytes than fish fed CTRL. Moreover, at 4 h, fish fed ST2.5, ST5, and ST10 showed a higher expression of interleukin 1β (*il1β*), while fish fed ST5 showed higher levels of ornithine decarboxylase (*odc)* than those fed CTRL. An upregulation of macrophage colony-stimulating factor 1 receptor 1 (*mcsf1r1*) and glutathione peroxidase (*gpx*) was also observed at 72 h in fish fed ST10 or ST5 and ST10 compared with CTRL, respectively.

**Discussion:**

In conclusion, incorporating up to 10% of the non-food fraction *S. ramosissima* in feed did not compromise seabass growth or immune status after 62 days, aligning with circular economy principles. However, *S. ramosissima* inclusion improved the leucocyte response and upregulated key immune-related genes in seabass challenged with an inactivated pathogen.

## 1 Introduction

Modern fish farming needs to cope with constraints such as limited availability and a high price of feed ingredients of a fish origin (1). In that sense, algae (2), insects (3), terrestrial animal by-products (4), and even plant protein sources, such as soybean, wheat, corn and others, are recognized as suitable alternatives (5). Despite being the most common alternative, the use of vegetable protein sources demands careful feed formulation with a detailed characterisation of the composition of vegetable ingredients. Such vegetable ingredients can incorporate some anti-nutritional factors (6, 7), present unbalanced essential amino acid profiles (8), and be lacking n-3 long chain poly-unsaturated fatty acids (9, 10). This could result in nutritionally unbalanced diets that might negatively impact fish physiological processes, culminating in lower growth performance (11) or poor health and welfare (7, 12, 13). As a countermeasure, plant protein sources are used in combination with the supplementation of specific crystalline amino acids to fulfill the animal requirement. Yet, at the moment, the use of such vegetable alternatives is seen as the most suitable response to the problem, which has resulted in the decreasing use of fishmeal and fish oil in fish feeds (14).

In that sense, plant protein sources, and particularly halophytes, can also be rich in valuable bioactive compounds (15), which reinforces their application as fish feedstuff. Halophytes, such as *Salicornia ramosissima* and *S. europaea*, are salt tolerant plants naturally found in saline soils; therefore, their cultivation does not compete for land with other vegetable crops (16, 17). In fact, future halophyte farms would have several environmental benefits, including: i) the utilisation of marginal and saline soils; ii) the bioremediation of effluents (e.g., aquaculture); iii) increased biodiversity; and iv) efficient CO_2_ sequestration through the expansion of aquatic biomass farms. Owing to the harsh environmental conditions, these plants produce high levels of bioactive compounds and radical-scavenging secondary metabolites (15), such as simple and complex sugars, quaternary ammonium compounds, polyols, and anti-oxidants (e.g., polyphenols, b-carotene, ascorbic acid, and ureides) (18). Giordano et al. (15) reviewed the current applications of the bioactive molecules found in *S. ramosissima* and *S. europaea* with health-promoting functions. Those compounds, despite varying with the particular habitat conditions, include anti-inflammatory, anti-bacterial and anti-oxidant properties, supported by the presence of hydroxycinnamic acids, phenolic acids, phenols, fatty acids, flavonoids, and others (19). Therefore, halophyte-derived protein or even whole plant incorporation in fish diets, at the expense of other plant protein sources, would present the added value of offering fish specific phytochemicals with immune-related functions. Moreover, the production of the secondary metabolites by industry is still a challenge that could limit the practical incorporation and evaluation of halophyte bioactive compounds in fish feeds. The principles of a circular economy can also be implemented in halophyte farming in which waste (i.e., the non-food fraction) is recovered to create value and new feed products.

In this agenda, the absence of studies assessing the capacity of the dietary incorporation of halophytes to modulate the antioxidant defenses, immune status, and inflammatory responses of farmed species (e.g., European seabass [*Dicentrarchus labrax*]) is noteworthy. Aquafeed optimisation, with the aim of producing a final formulation that can meet animal nutritional requirements but also present an additional health benefit, is a current approach. These feeds are usually known as functional diets.

The present study was designed to assess the effect of the inclusion of the non-food fraction of *S. ramosissima* in European seabass diets, focusing on their health condition after 62 days of feeding. The inflammatory response to inactivated *Photobacterium damselae* subsp. *piscicida* (*Phdp*) was also evaluated at the end of the feeding period.

## 2 Materials and methods

### 2.1 Heat inactivation of *Phdp*

The *Phdp* strain PP3, generously provided by Dr. Ana do Vale from the Institute for Molecular and Cell Biology at the University of Porto, Portugal, was originally isolated from yellowtail (*Seriola quinqueradiata*; Japan) by Dr. Andrew C. Barnes at the Marine Laboratory in Aberdeen, UK (20–22). Bacterial cultures were maintained at a standard temperature of 22°C in either tryptic soy broth (TSB) or tryptic soy agar (TSA), both sourced from Difco Laboratories. These media were supplemented with NaCl to achieve a final concentration of 1% (w/v) for TSB (designated as TSB-1) or TSA (designated as TSA-1). Long-term storage was conducted at −80°C in TSB-1 enriched with 15% (v/v) glycerol.

As detailed by do Vale et al. (20), for the preparation of the inoculum for fish peritoneal cavity injection, bacteria from the stock were cultured on TSA-1 for 48 h at 22°C. Subsequently, the culture was inoculated into TSB-1 and incubated overnight at 22°C with continuous shaking at 100 rpm. Exponentially growing bacteria were harvested by centrifugation at, 3500 × *g* for 30 min, resuspended in sterile Hank’s balanced salt solution (HBSS), and adjusted to a concentration of 3.4 × 10^5^ colony-forming units (cfu) ml^−1^. To ensure the absence of viable bacteria, the bacterial suspension underwent heat exposure at 60°C for 2 h. Confirmation of bacterial viability loss post-heat exposure was verified by plating the resulting cultures on TSA^-1^ plates, with the absence of any observable bacterial growth.

### 2.2 Halophyte biomass and experimental diets

Fresh *S. ramosissima* was collected from Praia da Areia Branca (Bunheiro-Murtosa, Portugal) in Summer, 2020. A total of 21.5 kg of *S. ramosissima* was collected. The edible fraction for humans (green tips) together with the roots were removed. The remaining non-food fraction was dried at Riasearch Lda. research facilities (Murtosa, Portugal), resulting in 6 kg of *S. ramosissima* dry biomass to be ground and incorporated into fish feeds.

Four diets were formulated and manufactured by Sparos Lda (Olhão, Portugal). The control diet (CTRL) was formulated to fulfil the known indispensable amino acid requirements of European seabass (23). The main ingredients were a blend of animal proteins of marine origin (40% fish and krill meals), a blend of plant proteins (47.3% soy protein concentrate, wheat gluten, corn gluten meal, and wheat meal), and a blend of fish and rapeseed oils (10.9%). At the expense of wheat meal, *S. ramosissima* whole non-food plant was incorporated at 3 different levels: 2.5, 5, and 10% of feed weight (ST2.5, ST5, and ST10, respectively). All diets were isoproteic (50% crude protein), isolipidic (16% crude fat), and isoenergetic (21 MJ/kg feed).

The main ingredients were ground (below 250 µm) using a micropulverizer hammer mill (SH1; Hosokawa Micron, B.V., Doetinchem, The Netherlands). Subsequently, the powdered ingredients and oils were blended in accordance with the specified formulation using a paddle mixer (RM90; Mainca, S.L., Granollers, Spain). All diets underwent production through temperature-controlled extrusion, yielding pellets of 1.5 mm in size, accomplished with a low-shear extruder (P55; Italplast, S.r.l., Parma, Italy). Following extrusion, each batch of feed was subjected to a drying process in a convection oven (OP 750-UF; LTE Scientifics, Oldham, UK) for a duration of 4 h at 45°C.

Bromatological analysis of experimental diets was performed following the Association of Official Analytical Chemists procedures (24). Briefly, dry matter was determined by drying samples at 105°C in an oven until constant weight; ash, through incineration at 450°C for 16 h in a muffle furnace; crude protein content (N x 6.25) using the Kjeldahl method after acid digestion using a Kjeltec digestion and distillation unit; and lipid content, by petroleum ether extraction (Soxtec HT System) and gross energy, through direct combustion in an adiabatic bomb calorimeter (PARR Instruments, Moline, IL, USA; PARR model, 1261).

The main ingredients and analysed proximate composition of the experimental diets are detailed by Marçal et al. (25).

### 2.3 Trial design

European seabass juveniles with average initial weight of 7.26 ± 0.06 g were obtained from Sonríonansa S.L. (Santander, Spain) and acclimated to Riasearch Lda. research facilities. Nine hundred and sixty fish were randomly distributed to 12 350 L tanks (80 individuals per tank) composed of an 18 m^3^ recirculating aquaculture system with a water renewal of 1 tank per hour. Water parameters were measured once a day using commercial probes and the temperature was maintained at 21.6 ± 0.2°C, dissolved oxygen at 6.4 ± 0.6 mg L^-1^, salinity at 18.2 ± 0.2 g L^-1^, pH at 7.5 ± 0.2, and nitrogen compounds below 0.1 mg L^-1^.

#### 2.3.1 Health status trial

To evaluate the effects of *S. ramosissima* inclusion in diets on the health performance of European seabass juveniles (starting weight 8.62 ± 0.63 g), a feeding trial was performed; each diet was distributed in triplicate tanks and fish were fed for 34 or 62 days. At the indicated times, fish were sorted and euthanized for tissue sampling. From five individuals per tank (15 per experimental group), blood was collected from the caudal vessels, plasma was isolated for the assessment of innate immune parameters, and the head kidney was sampled to analyse the expression of key immune-related genes through RT-qPCR. Moreover, blood was also collected from three individuals per tank (9 per treatment) and used for the haematological profile evaluation. Additionally, body weight (average final weight 43.12 ± 3.19 g) and peritoneal exudates from three fish per tank (9 per treatment) were collected at 62 days.

#### 2.3.2 Inflammatory trial

With the aim of assessing the modulatory role of whole *S. ramosissima* dietary inclusion in inflammatory processes, the inflammatory response was further studied in a time-course fashion. At the end of the 62-day feeding period, 12 fish per tank were re-allocated in a similar recirculation system at Interdisciplinary Centre of Marine and Environmental Research facilities (CIIMAR, Matosinhos, Portugal), and the individuals were submitted to an inflammatory challenge with heat-inactivated *Phdp* strain PP3 (3.4 × 10^5^ CFU ml^-1^). After that, three individuals from each tank (nine per treatment) were sampled for blood, head kidney, and peritoneal exudates at 4, 24, 48, and 72 h after the challenge period to assess cell proliferation, plasma immune parameters, and immune-related genes.

### 2.4 Haematological profile and peritoneal exudates

The haematological profile encompassed total white (WBC) and red (RBC) blood cell counts, along with haematocrit (Ht) and haemoglobin (Hb; SPINREACT Kit; ref., 1001230, Spain). Mean corpuscular volume (MCV), mean corpuscular haemoglobin (MCH), and mean corpuscular haemoglobin concentration (MCHC) were subsequently calculated according to the methodology outlined by Machado et al. (22).

Following blood collection, blood smears were performed, air-dried, and fixed with formol-ethanol (10% of 37% formaldehyde in absolute ethanol). Peroxidase detection facilitated the identification of neutrophils. Wright’s stain (Haemacolor; Merck) was then applied to the blood smears, which were examined at, 1000× magnification. A minimum of 200 leucocytes were counted and categorized as thrombocytes, lymphocytes, monocytes, and neutrophils. The absolute value (× 10^4^ ml^−1^) of each cell type was calculated based on the total blood WBC counts

Peritoneal cells were exclusively collected from fish sampled after 62 days of feeding and at 4, 24, and 48 h post-inflammation as part of a time-course analysis. After fish anaesthesia and bleeding from the caudal vessel, 2 ml of cold Hank’s balanced salt solution (HBSS) supplemented with 30 units of heparin ml^−1^ was inoculated into the peritoneal cavity. Subsequently, the peritoneal area was gently massaged to disperse peritoneal cells in the injected HBSS. Then, the injected HBSS containing suspended cells was collected, and total peritoneal leucocyte counts were conducted using a haemocytometer. Cytospin preparations were made with a THARMAC Cellspin apparatus and stained as described above for blood smears. Lymphocyte, macrophage, and neutrophil concentrations in the peritoneal exudates were determined by assessing the percentage of each cell type after counting a minimum of 200 cells per slide. The concentration (×10^4^ ml^−1^) of each leucocyte type was also calculated.

### 2.5 Plasma humoral parameters

#### 2.5.1 Lysozyme

Lysozyme activity was assessed via a turbidimetric assay following the protocol outlined by Costas et al. (26). A *Micrococcus lysodeikticus* solution (0.5 mg ml^−1^, 0.05 M sodium phosphate buffer, pH 6.2) was prepared. In triplicate, 15 µl of plasma was combined with 250 µl of the prepared suspension in a microplate, resulting in a final volume of 265 µl. The reaction, conducted at 25°C, was monitored by measuring absorbance at 450 nm after 0.5 and 4.5 min using a SynergyHT microplate reader. A standard curve was established using serially diluted lyophilized hen egg white lysozyme (Sigma-Aldrich) in sodium phosphate buffer (0.05 M, pH 6.2), facilitating the calculation of lysozyme levels in the sample based on the standard curve formula.

#### 2.5.2 Peroxidase

Total peroxidase activity in plasma was measured following the procedure described by Quade and Roth (27). In triplicate wells, 15 µl of plasma was diluted with 135 µl of HBSS without Ca^+2^ and Mg^+2^ in flat-bottomed 96-well plates. Subsequently, 50 µl of 20-mM 3,3′,5,5′-tetramethylbenzidine hydrochloride (TMB; Sigma) and 50 µl of 5-mM H_2_O_2_ were added. After 2 min, the colour-change reaction was halted by adding 50 µl of 2 M sulfuric acid. The optical density was measured at 450 nm in a SynergyHT microplate reader. Wells without plasma served as blanks and peroxidase activity (units ml^−1^ plasma) was determined, with one unit defined as the amount producing an absorbance change of 1 optical density (OD).

#### 2.5.3 Anti-proteases

Anti-protease activity was determined as described by Machado et al. (22). Plasma (10 µl) was incubated with an equal volume of trypsin solution (5 mg ml^−1^ in NaHCO3, 5 mg ml^−1^, pH 8.3) for 10 min at 22°C. Subsequently, phosphate buffer (100 ml; NaH2PO4, 13.9 mg ml^−1^, pH 7.0) and azocasein (125 ml; 20 mg ml^−1^ in NaHCO3, 5 mg ml^−1^, pH 8.3) were added, followed by incubation for 1 h at 22°C. The reaction was stopped with trichloroacetic acid (250 ml), and the resulting mixture was centrifuged. Afterward, the supernatant (100 ml) was transferred to a 96-well plate containing NaOH (100 ml; 40 mg ml−1 per well). Optical density at 450 nm was measured in a SynergyHT microplate reader. The percentage inhibition of trypsin activity compared with the reference sample was calculated. All analyses were duplicated.

#### 2.5.4 Bactericidal activity

Bactericidal activity was assessed using *Phdp* strain PP3. Bacteria were cultured in tryptic soy broth (TSB) (Difco Laboratories) with NaCl (2% w/v) (TSB-2). Exponentially growing bacteria were resuspended in sterile HBSS and adjusted to 1 × 10^6 cfu ml^−1^. Plasma bactericidal activity was determined following the method described by Machado et al. (22). Plasma (20 µl) was added to duplicate wells of a U-shaped 96-well plate, with HBSS used in some wells as a positive control. To each well, 20 µl of Phdp (1 × 10^6^ cfu ml^−1^) was added, and the plate was incubated for 2.5 h at 25°C. MTT (25 µl; 1 mg ml^−1^; Sigma) was added to each well, and after 10 min at 25°C, the absorbance was measured at 560 nm in a SynergyHT microplate reader. Non-viable bacteria were expressed as a percentage, calculated from the difference between the dissolved formazan in samples and that formed in the positive controls (100%).

### 2.6 Gene expression

Total RNA isolation from the head kidney and subsequent DNase treatment were conducted using an NZY Total RNA Isolation Kit (MB13402, NZYTech, Portugal), following the manufacturer’s guidelines. First-strand cDNA synthesis was performed using an NZY First-Strand cDNA Synthesis Kit (MB125, NZYTech, Portugal), adhering to the recommended protocols. The design of primers, determination of efficiency values, and quantitative PCR assays were carried out. Primer efficiency was calculated based on a series dilution of the head kidney cDNA pool. The primer efficiency and DNA amplification processes were executed using a CFX384 Touch Real-Time PCR Detection System, employing specific primers for genes chosen for their involvement in immune responses (refer to **Table 1**). Accession numbers, efficiency values, annealing temperatures, product lengths, and primer sequences are detailed in **Table 1**. Additionally, a melting curve analysis was performed to ensure the absence of primer dimers. The standard cycling conditions comprised an initial denaturation step of 10 min at 95°C, followed by 40 cycles of two steps (95°C for 15 s and primer annealing temperature for 1 min). Subsequently, there was a 1-min period at 95°C, followed by 35 s at the primer annealing temperature, concluding with 0.5 s at 95°C. All reactions were conducted as technical duplicates. To normalise the expression of target genes, the average expression of *Dicentrarchus labrax* Eukaryotic small ribosomal subunit 40 (*40s*) and Elongation factor 1-alpha (*ef1α*) was employed.

**Table 1:** Forward and reverse primers for real-time PCR.

Acronym	Gene name	GenBank ID	Eff^1^	AT^2^	Product lenght^3^	Primer sequence (5′-3′)	
*40s*	Eukaryotic small ribosomal subunit 40	HE978789.1	108.78	60	79	F-	TGATTGTGACAGACCCTCGTG
						R-	CACAGAGCAATGGTGGGGAT
*ef1α*	Elongation factor 1-alpha	AJ866727.1	96.45	57	144	F-	AACTTCAACGCCCAGGTCAT
						R-	CTTCTTGCCAGAACGACGGT
*il1β*	Interleukin 1 β	AJ269472.1	96.7	57	105	F-	AGCGACATGGTGCGATTTCT
						R-	CTCCTCTGCTGTGCTGATGT
*il10*	Interleukin 10	AM268529.1	116	55	164	F-	ACCCCGTTCGCTTGCCA
						R-	CATCTGGTGACATCACTC
*mmp9*	Matrix-metalloproteinase 9	FN908863.1	98.44	57	166	F-	TGTGCCACCACAGACAACTT
						R-	TTCCATCTCCACGTCCCTCA
*nf-κB*	Nuclear Factor Kappa B	DLAgn_00239840	113.28	55	136	F-	GCTGCGAGAAGAGAGGAAGA
						R-	GGTGAACTTTAACCGGACGA
*mcsf1r1*	Macrophage colony-stimulating factor 1 receptor 1	DLAgn_00109630	83	62	200	F-	ATGTCCCAACCAGACTTTGC
						R-	GGCTCATCACACACTTCACC
*cd8β*	Cluster of differentiation 8 β chain	DLAgn_00090370	113.81	55	223	F-	CGGAACCCAAAAGGCCAAAG
						R-	TAGGCTGTAGATGCAGTGCT
*tlr9*	Toll-like receptor 9	KX399289	115.17	55	100	F-	TCTTGGTTTGCCGACTTCTTGCGT
						R-	TACTGTTGCCCTGTTGGGACTCTGG
*myd88*	Myeloid differentiation primary response protein	KM225785	116.47	57	71	F-	CCAATTCAGGTTGATGAGGTTGACA
						R-	TCCTCCAGGGTGATACCAATCC
*odc*	Ornithine decarboxylase	KM225771	113.18	60	69	F-	GGGCTGTAGTTATGACACTGGCATCC
						R-	GCTGAATCTCCATCTTGCTTGCACAGT
*gpx*	Glutathione peroxidase	DLAgn_00004360	107.59	60	150	F-	ACCCTCTGTGGAAGTGGATG
						R-	TGCTACTTACGCTGGGATCA
*sod*	Superoxide dismutase	CX660893.1	103.03	55	71	F-	GGAGAGTGATTCAGCCCCTG
						R-	GGAAACCATGCTCACCAGGA

^1^The efficiency of PCR reactions was calculated from serial dilutions of tissue RT reactions in the validation procedure.

^2^Annealing temperature (°C).

^3^Amplicon (bp).

### 2.7 Data analyses

All results are presented as mean ± standard deviation (mean ± SD). Data were assessed for normality and homogeneity of variance and, if necessary, transformed before statistical analysis. Two-way ANOVA, with diet and time as factors, was employed, followed by the Tukey *post-hoc* test to identify differences among treatments. In the time-course data analysis, the sampling point at 62 days was designated as time 0 h to evaluate the activation of inflammatory mechanisms.

## 3 Results

### 3.1 Immune status and growth

**Figure 1:**
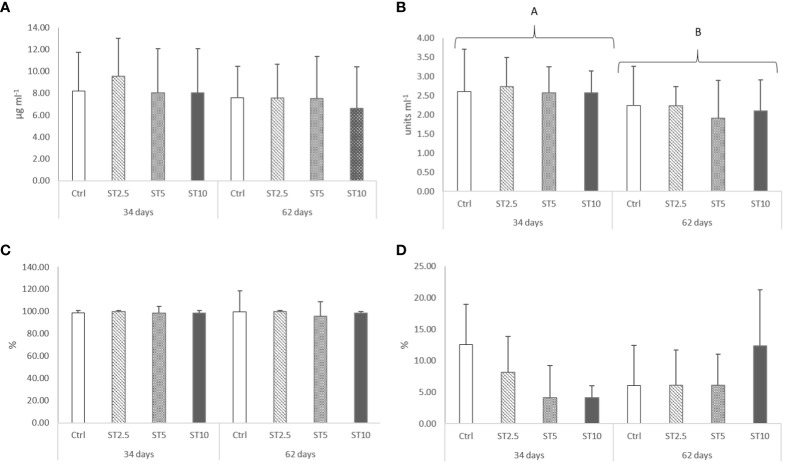
Plasma **(A)** lysozyme (p-value ≥ 0.05), **(B)** peroxidase (p-value = 0.046), **(C)** anti-protease activity (p-value ≥ 0.05) and **(D)** bactericidal activity (p-value ≥ 0.05) in European seabass fed dietary treatments for 34 and 62 days. Data are mean ± SD (n=9). Two-way ANOVA (p ≤ 0.05).

No significant differences were observed in the growth (average final weight, 43.12 ± 3.19 g), survival (94–97%), haematological profile (**Table 2**), peripheral differential leucocyte numbers (**Table 3**), and plasma humoral parameters (**Figure 1**) of fish fed for 34 or 62 days with any of the *S. ramosissima* inclusion levels compared with those fed the control diet.

**Table 2:** Peripheral white blood cells (WBC), red blood cells (RBC), haematocrit (HT), haemoglobin (HG), mean corpuscular haemoglobin (MHC), mean corpuscular volume (MCV), and mean corpuscular haemoglobin concentration (MCHC) in European seabass fed dietary treatments for 34 and 62 days.

Parameters	Dietary treatments																								Two-way ANOVA			
	34 days												62 days													Time	Diet	Time × diet
	CTRL			ST2.5			ST5			ST10			CTRL			ST2.5			ST5			ST10						
WBC, × 10^4^ µl^-1^	9.27	±	1.89	8.94	±	2.03	8.62	±	1.44	2.50	±	1.39	7.29	±	1.26	7.60	±	1.07	7.42	±	1.31	7.53	±	0.81	WBC	ns	ns	ns
RBC, × 10^6^ µl^-1^	2.77	±	0.42	2.60	±	0.32	2.62	±	0.58	0.35	±	0.37	2.91	±	0.44	3.35	±	0.87	3.47	±	1.07	3.02	±	0.66	RBC	ns	ns	ns
HT, %	35.38	±	3.87	34.78	±	1.69	32.67	±	2.91	34.56	±	2.50	35.44	±	3.30	35.33	±	4.40	32.71	±	8.78	37.00	±	3.68	HT	ns	ns	ns
HG, g dl^-1^	1.54	±	0.30	1.46	±	0.24	1.50	±	0.29	1.50	±	0.35	1.46	±	0.74	1.51	±	0.59	1.49	±	0.49	1.47	±	0.35	HG	ns	ns	ns
MCH, pg cell^-1^	5.78	±	1.83	5.66	±	0.95	5.91	±	1.29	6.95	±	2.06	5.33	±	3.12	4.96	±	2.77	4.94	±	2.57	5.11	±	1.56	MCH	ns	ns	ns
MCV, µ m^3^	129.24	±	15.52	135.88	±	19.93	131.44	±	35.03	129.58	±	18.17	124.03	±	16.21	113.10	±	32.83	91.14	±	19.47	128.03	±	28.34	MCV	ns	ns	ns
MCHC, g 100 ml^-1^	4.54	±	1.13	4.21	±	0.77	4.57	±	0.59	5.33	±	1.15	4.14	±	2.10	4.40	±	1.87	5.14	±	3.53	4.03	±	1.11	MCHC	ns	ns	ns

Data are mean ± SD (n=9). One-way ANOVA (p ≤ 0.05). ns, not significant.

**Table 3:** Peripheral differential leucocyte counting in European seabass fed dietary treatments for 34 and 62 days.

Parameters		Dietary treatments																								Two-way ANOVA			
		34 days												62 days													Time	Diet	Time × diet
		CTRL			ST2.5			ST5			ST10			CTRL			ST2.5			ST5			ST10						
Neutrophils	× 10^4^ µl^-1^	0.18	±	0.11	0.14	±	0.08	0.14	±	0.12	0.13	±	0.07	0.12	±	0.07	0.20	±	0.10	0.09	±	0.09	0.11	±	0.10	Neutrophils	ns	ns	ns
Monocytes		0.10	±	0.08	0.11	±	0.06	0.14	±	0.13	0.10	±	0.07	0.11	±	0.06	0.12	±	0.09	0.11	±	0.11	0.09	±	0.06	Monocytes	ns	ns	ns
Lymphocytes		3.65	±	1.06	3.10	±	0.92	3.22	±	0.95	2.54	±	0.77	1.85	±	0.70	1.49	±	0.57	1.42	±	0.66	1.52	±	0.39	Lymphocytes	<0.001	ns	ns
Thrombocytes		5.39	±	1.73	5.65	±	1.51	5.21	±	0.92	5.50	±	1.34	5.25	±	0.79	5.84	±	0.89	5.16	±	2.05	5.83	±	0.63	Thrombocytes	ns	ns	ns

Data are mean ± SD (n=9). Two-way ANOVA (p ≤ 0.05). If the interaction was significant, a Tukey post-hoc test was used to identify differences in the experimental treatments. ns, not significant.

**Figure 2:**
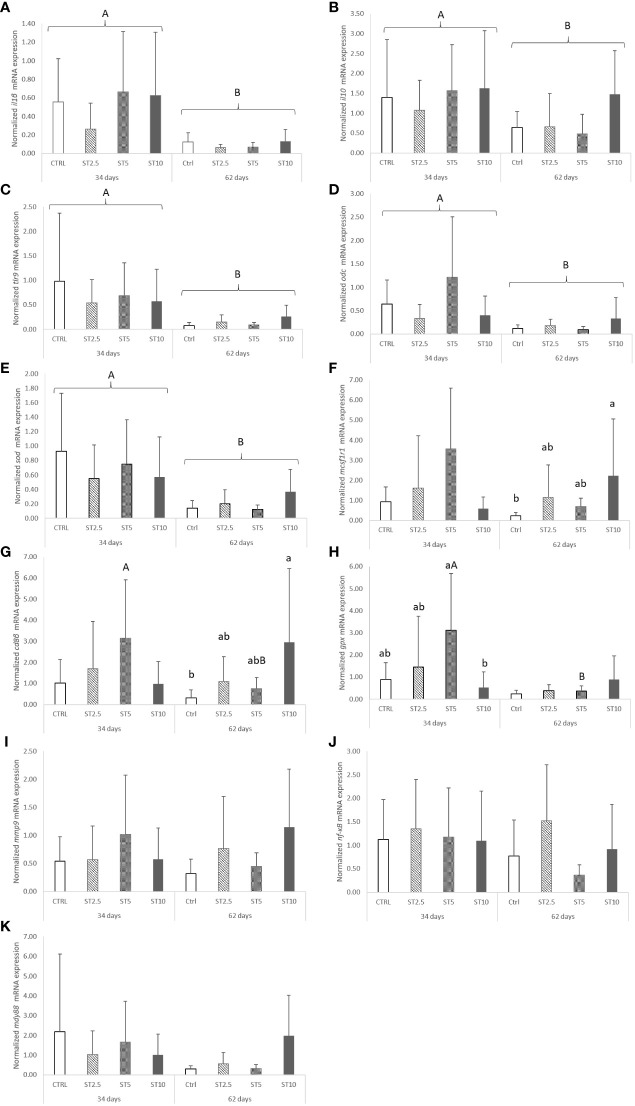
Quantitative expression of **(A)** Interleukin 1 β (p-value < 0.001), **(B)** Interleukin 10 (p-value =0.037), **(C)** Matrix-metalloproteinase 9 (p-value ≥ 0.05), **(D)** Nuclear Factor Kappa B (p-value ≥ 0.05), **(E)** Macrophage colony-stimulating factor 1 receptor 1 (p-value = 0.028), **(F)** Cluster of differentiation 8 beta chain (p-value= 0.033), **(G)** Toll-like receptor 9 (p-value = 0.001), **(H)** Myeloid differentiation primary response protein (p-value ≥ 0.05), **(I)** Ornithine decarboxylase (p-value= 0.002), **(J)** Glutathione peroxidase (p-value = 0.027), and **(K)** Superoxide dismutase (p-value < 0.001) in the head kidney of European seabass fed the dietary treatments for 34 and 62 days. Data are mean ± SD (n = 9). P-values from two-way ANOVA (p ≤ 0.05). If the interaction was significant, a Tukey *post-hoc* test was used to identify differences in the experimental treatments. Different capital letters indicate differences among times, while lower case letters represent differences among diets.

However, some changes were observed related to feeding time in peripheral cells and in the head kidney mRNA expression levels of immune-related genes. In particular, a decrease in peripheral lymphocyte numbers was observed in European seabass fed for 62 days compared with those fed for 34 days (**Table 3**). Likewise, the mRNA expression levels of *il1β, il10, tlr9, odc*, and *sod* also decreased in fish fed for 62 days compared with those fed for 34 days (**Figures 2A–E**). Nonetheless, seabass fed ST10 showed an upregulation of *mcsf1r1* (**Figure 2F**) and *cd8β* (**Figure 2G**) compared with the CTRL-fed fish after 62 days of feeding. Moreover, *gpx* mRNA expression levels decreased in seabass fed ST10 compared with those fed ST5 after 34 days of feeding (**Figure 2H**). Finally, feeding time had an impact on the expression levels of *cd8β* and *gpx* in fish fed ST5, with a decrease in expression from 34 to 62 days of feeding. No differences were observed in mmp9 (**Figure 2I**), nf-κB (**Figure 2J**) and myd88 (**Figure 2K**), expressions.

### 3.2 Inflammatory response

**Figure 3:**
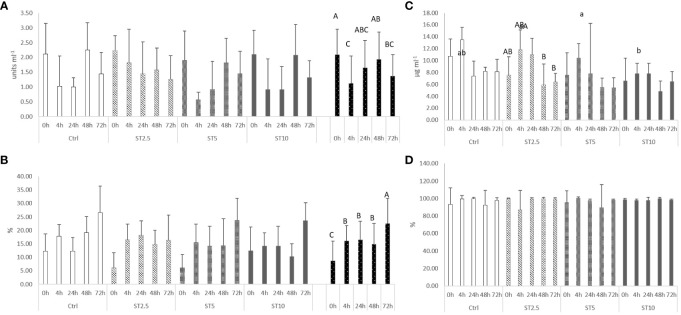
Plasma **(A)** lysozyme (p-value < 0.004), **(B)** peroxidase (p-value < 0.001), **(C)** anti-protease activity, and **(D)** bactericidal activity (p-value < 0.001) in European seabass fed dietary treatments at 63 days (0 h), 4, 24, 48 and 72 h after inflammatory stimulus. Data are mean ± SD (n=9). Two-way ANOVA (p ≤ 0.05). If the interaction was significant, a Tukey *post-hoc* test was used to identify differences in the experimental treatments. Different capital letters indicate differences among times, while lower case letters represent differences among diets.

**Figure 4:**
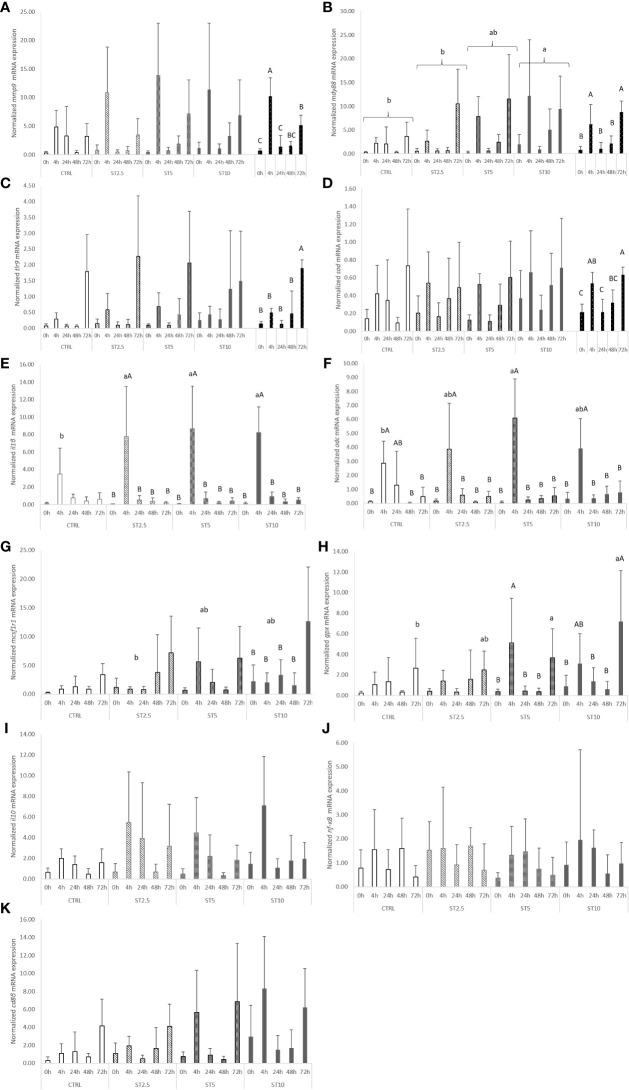
Quantitative expression of **(A)** Interleukin 1 β (p-value < 0.011), **(B)** Interleukin 10 (p-value < 0.05), **(C)** Matrix-metalloproteinase 9 (p-value < 0.001), **(D)** Nuclear Factor Kappa B (p-value > 0.05), **(E)** Macrophage colony-stimulating factor 1 receptor 1 (p-value = 0.036), and **(F)** Cluster of differentiation 8 beta chain (p-value < 0.05) in the head kidney of European seabass fed the dietary treatments at 63 days (0 h), 4, 24, 48 and 72 h after inflammatory stimulus. Data are mean ± SD (n = 9). P-values from two-way ANOVA (p ≤ 0.05). If the interaction was significant, a Tukey *post-hoc* test was used to identify differences in the experimental treatments. Different capital letters indicate differences among times, while lower case letters represent differences among diets. Quantitative expression of **(G)** Toll-like receptor 9 (p-value < 0.001), **(H)** Myeloid differentiation primary response protein (diet p-value = 0.001; sampling time p-value < 0.001), **(I)** Ornithine decarboxylase (p-value = 0.030), **(J)** Glutathione peroxidase (p-value = 0.007) and **(K)** Superoxide dismutase (p-value < 0.001) in the head kidney of European seabass fed the dietary treatments at 63 days (0 h), 4, 24, 48 and 72 h after inflammatory stimulus. Data are mean ± SD (n = 9). P-values from two-way ANOVA (p ≤ 0.05). If the interaction was significant, a Tukey *post-hoc* test was used to identify differences in the experimental treatments. Different capital letters indicate differences among times, while lower case letters represent differences among diets.

Most changes were observed among the sampling times after the onset of inflammation. On the one hand, a general decrease in several haematological parameters, such as total RBC, HT, HG, MCV, MCH, and MCHC (**Table 4**), accompanied by a decrease in plasma peroxidase activity (**Figure 3A**) and peripheral lymphocyte concentration at 4 h (**Table 5**), was observed. On the other hand, an increase in peripheral and peritoneal WBC numbers at 4 and 24 h (**Table 5**), as well as plasma bactericidal activity (**Figure 3B**), was observed in response to inflammation. Moreover, circulating neutrophils and monocytes also increased at 24 h (**Table 5**). The increase in inflammatory indicators was also observed with the gene expression analysis, with the upregulation of *mmp9* (**Figure 4A**) and *myd88* (**Figure 4B**) at 4 and 72 h compared with 0 h and 0, 4, 24 and 48 h, respectively. Additionally, *tlr9* (**Figure 4C**) and *sod* (**Figure 4D**) expression increased 72 h after inflammatory induction compared with all the remaining sampling times.

Table 4Peripheral white blood cells (WBC), red blood cells (RBC), haematocrit (HT), haemoglobin (HG), mean corpuscular haemoglobin (MHC), mean corpuscular volume (MCV), mean corpuscular haemoglobin concentration (MCHC), and peritoneal leucocytes in European seabass fed dietary treatments at 62 days (0h), 4, 24, 48, and 72 h after inflammatory stimulus.

**Table:** 

Parameters	Dietary treatments																							
	T0h (62 days)												4 h											
	CTRL			ST2.5			ST5			ST10			CTRL			ST2.5			ST5			ST10		
WBC, × 10^4^ µl^-1^	7.29	±	1.26	7.60	±	1.07	7.42	±	1.31	7.53	±	0.81	5.77	±	1.16	5.36	±	1.64	5.36	±	1.09	6.38	±	1.55
RBC, × 10^6^ µl^-1^	2.91	±	0.44	3.35	±	0.87	3.47	±	1.07	3.02	±	0.66	2.65	±	0.16	2.88	±	0.46	2.88	±	0.34	2.66	±	0.67
HT, %	35.44	±	3.30	35.33	±	4.40	32.71	±	8.78	37.00	±	3.68	31.22	±	2.78	24.25	±	3.40	24.25	±	3.70	27.56	±	4.37
HG, g dl^-1^	1.46	±	0.74	1.51	±	0.59	1.49	±	0.49	1.47	±	0.35	1.36	±	0.59	1.35	±	0.31	1.35	±	0.66	1.14	±	0.38
MCH, pg cell^-1^	5.33	±	3.12	4.96	±	2.77	4.94	±	2.57	5.11	±	1.56	5.19	±	2.26	4.67	±	1.13	4.67	±	2.18	4.99	±	3.22
MCV, µ m^3^	124.03	±	16.21	113.10	±	32.83	91.14	±	19.47	128.03	±	28.34	118.57	±	14.67	85.94	±	25.23	85.94	±	9.72	107.61	±	19.61
MCHC, g 100 ml^-1^	4.14	±	2.10	4.40	±	1.87	5.14	±	3.53	4.03	±	1.11	4.35	±	1.82	5.23	±	1.11	5.23	±	2.62	4.43	±	2.23
Peritoneal leucocytes, × 10^4^ µl^-1^	1.73	±	0.77 C	0.88	±	0.36 C	1.02	±	0.28 C	0.76	±	0.35 B	6.17	±	1.50 bAB	7.47	±	2.07 abA	7.51	±	3.20 abA	9.77	±	2.96 aA
Parameters	Dietary treatments																							
	24 h												48 h											
	CTRL			ST2.5			ST5			ST10			CTRL			ST2.5			ST5			ST10		
WBC, × 10^4^ µl^-1^	8.94	±	2.23	7.14	±	2.79	8.39	±	2.17	7.22	±	2.20	7.29	±	2.30	6.50	±	2.11	6.51	±	1.43	6.44	±	1.21
RBC, × 10^6^ µl^-1^	2.90	±	0.44	2.90	±	0.46	2.95	±	0.46	2.64	±	0.58	3.04	±	0.37	3.03	±	0.46	2.78	±	0.54	2.76	±	0.41
HT, %	31.00	±	3.00	28.56	±	2.65	29.33	±	5.33	29.56	±	3.34	32.88	±	3.14	32.29	±	1.98	31.29	±	2.85	31.63	±	2.29
HG, g dl^-1^	1.15	±	0.33	1.29	±	0.48	1.24	±	0.36	1.07	±	0.19	1.01	±	0.21	1.06	±	0.18	0.93	±	0.32	1.26	±	0.34
MCH, pg cell^-1^	4.02	±	1.08	4.55	±	2.29	4.32	±	1.41	4.23	±	0.98	3.33	±	0.70	3.57	±	0.68	3.48	±	1.18	4.54	±	1.00
MCV, µ m^3^	108.99	±	15.93	101.95	±	27.70	100.93	±	18.64	117.23	±	28.79	109.41	±	8.83	102.06	±	12.56	114.94	±	14.37	115.27	±	18.00
MCHC, g 100 ml^-1^	3.74	±	1.24	4.57	±	1.56	4.30	±	1.26	3.65	±	0.66	3.13	±	0.69	3.38	±	0.49	3.01	±	1.21	4.00	±	1.15
Peritoneal leucocytes, × 10^4^ µl^-1^	7.02	±	2.02 A	7.47	±	1.84 A	7.34	±	2.24 A	8.48	±	2.62 A	4.76	±	1.44 ABC	4.77	±	1.38 AB	5.27	±	1.96 AB	3.60	±	1.72 B

**Table:** 

Parameters	Dietary treatments											
	72 h											
	CTRL			ST2.5			ST5			ST10		
WBC, × 10^4^ µl^-1^	6.76	±	2.00	8.39	±	2.21	6.31	±	1.75	8.03	±	2.69
RBC, × 10^6^ µl^-1^	2.47	±	0.39	2.72	±	0.35	2.61	±	0.31	2.67	±	0.30
HT, %	33.67	±	4.85	30.00	±	3.84	29.57	±	5.55	30.71	±	3.19
HG, g dl^-1^	0.84	±	0.15	0.91	±	0.19	1.03	±	0.23	0.95	±	0.13
MCH, pg cell^-1^	3.45	±	0.84	3.34	±	0.58	3.95	±	0.85	3.60	±	0.57
MCV, µ m^3^	135.09	±	12.74	113.85	±	13.74	110.42	±	10.11	118.55	±	13.70
MCHC, g 100 ml^-1^	2.53	±	0.51	2.97	±	0.66	3.65	±	0.85	3.21	±	0.60
Peritoneal leucocytes, × 10^4^ µl^-1^	3.11	±	1.66 BC	2.72	±	0.69 B	3.94	±	1.10 B	2.31	±	1.11 B
Two-way ANOVA.												
	Time	Diet	Time × diet	Time					Dietary treatments			
				T0h (62 days)	4 h	24 h	48 h	72 h	CTRL	ST2.5	ST5	ST10
WBC	<0.001	ns	ns	AB	CD	A	BC	AC	–	–	–	–
RBC	< 0.001	ns	ns	A	B	B	AB	B	–	–	–	–
HT	< 0.001	0.005	ns	A	C	C	B	B	a	ab	b	ab
HG	<0.001	ns	ns	A	AB	BC	BC	C	–	–	–	–
MCH	0.002	ns	ns	A	AB	ABC	BC	C	–	–	–	–
MCV	0.046	<0.001	ns	AB	B	AB	AB	A	a	ab	b	a
MCHC	<0.001	ns	ns	ABC	A	ABCD	CD	D	–	–	–	–
Peritoneal leucocytes	<0.001	ns	0.008	B	A	A	C	D	–	–	–	–

Data are mean ± SD (n=9). Two-way ANOVA (p ≤ 0.05). If the interaction was significant, a Tukey post-hoc test was used to identify differences in the experimental treatments. Different capital letters indicate differences among times, while lower case letters represent differences among diets. ns, not significant.

Table 5Peripheral cell count in European seabass fed dietary treatments at 62 days (0h), 4, 24, 48 and 72 h after inflammatory stimulus.

**Table:** 

Parameters		Dietary treatments																							
		T0h (62 days)												4 h											
		CTRL			ST2.5			ST5			ST10			CTRL			ST2.5			ST5			ST10		
Neutrophils	× 10^4^ µl^-1^	0.11	±	0.07	0.20	±	0.10	0.09	±	0.09	0.11	±	0.10	1.0	±	0.4	1.4	±	0.6	0.8	±	0.3	1.3	±	0.0
Monocytes		0.11	±	0.06	0.12	±	0.09	0.11	±	0.11	0.09	±	0.06	0.1	±	0.1	0.1	±	0.2	0.0	±	0.0	0.0	±	0.0
Lymphocytes		1.85	±	0.70	1.49	±	0.57	1.42	±	0.66	1.52	±	0.39	0.1	±	0.1	0.2	±	0.2	0.1	±	0.1	0.1	±	0.1
Thrombocytes		5.25	±	0.79	5.84	±	0.89	5.16	±	2.05	5.83	±	0.63	4.6	±	1.1	5.5	±	1.2	4.4	±	0.8	5.0	±	1.3
Parameters		Dietary treatments																							
		24 h												48 h											
		CTRL			ST2.5			ST5			ST10			CTRL			ST2.5			ST5			ST10		
Neutrophils	× 10^4^ µl^-1^	2.11	±	1.16	1.35	±	0.70	1.74	±	1.01	2.18	±	1.14	0.51	±	0.27	0.36	±	0.24	0.42	±	0.24	0.47	±	0.24
Monocytes		0.12	±	0.09	0.13	±	0.11	0.22	±	0.15	0.13	±	0.09	0.08	±	0.05	0.05	±	0.04	0.08	±	0.04	0.05	±	0.04
Lymphocytes		0.20	±	0.54	0.20	±	0.71	1.60	±	0.69	1.28	±	0.46	2.05	±	0.81	1.57	±	0.91	1.43	±	0.39	1.58	±	0.57
Thrombocytes		5.12	±	1.83	5.51	±	2.02	4.89	±	1.61	3.72	±	1.35	4.76	±	1.40	4.52	±	1.23	4.58	±	1.18	4.43	±	0.75

**Table:** 

Parameters			Dietary treatments											
			72 h											
			CTRL			ST2.5			ST5			ST10		
Neutrophils		× 10^4^ µl^-1^	0.21	±	0.15	0.14	±	0.11	0.22	±	0.11	0.38	±	0.26
Monocytes			0.07	±	0.04	0.07	±	0.06	0.07	±	0.05	0.11	±	0.07
Lymphocytes			2.03	±	0.72	2.03	±	0.96	1.06	±	0.30	1.50	±	0.52
Thrombocytes			6.19	±	2.23	4.60	±	1.71	4.98	±	1.56	6.10	±	2.29

**Table:** 

Two-way ANOVA.								
	Time	Diet	Time × diet	Time				
				T0h (62 days)	4 h	24 h	48 h	72 h
Neutrophils	<0.001	ns	ns	C	B	A	C	C
Monocytes	<0.001	ns	ns	AB	B	A	B	B
Lymphocytes	<0.001	ns	ns	A	B	A	A	A
Thrombocytes	ns	ns	ns	–	–	–	–	–

Data are mean ± SD (n=9). Two-way ANOVA (p ≤ 0.05). If the interaction was significant, a Tukey post-hoc test was used to identify differences in the experimental treatments. Different capital letters indicate differences among times. ns, not significant.

Regarding the effect of *S. ramosissima* dietary inclusion on the inflammatory response, seabass fed ST5 showed a decrease in HT and MCV values compared with those fed CTRL and ST10, respectively. By contrast, fish fed ST10 showed lower plasma lysozyme levels than their counterparts fed ST2.5 and ST5 at 24 h following injection (**Figure 3C**), while at 4 h after the inflammatory stimulus, seabass fed ST10 presented a higher concentration of peritoneal leucocytes than fish fed CTRL (**Table 4**).

The expression of pro-inflammatory genes coding for IL1β, MCSF1R, MYD88, ODC, and GPX also increased in seabass fed diets with halophyte inclusion in response to inflammation. Fish fed ST2.5, ST5, and ST10 showed a higher expression of *il1β* than those fed CTRL 4 h after i.p. injection (**Figure 4E**), while at the same sampling time (4 h), seabass fed ST5 showed higher *odc* than those fed CTRL (**Figure 4F**). An upregulation of *mcsf1r1* (**Figure 4G**) and *gpx* (**Figure 4H**) was also observed at 72 h in fish fed ST10 or ST5 and ST10 compared with CTRL, respectively. Finally, and regardless of sampling time, the expression of MYD88 (**Figure 4B**) augmented in ST10 compared with CTRL and ST2.5.

## 4 Discussion

The reasoning behind the dietary inclusion of halophytes (i.e., *S. ramosissima*) goes beyond its use as an alternative protein source in the agenda of aquafeed optimisation as its dietary incorporation could also present the added value of offering fish specific phytochemicals with immune-related benefits. Hence, the effect of *S. ramosissima* inclusion (non-food fraction) in diets for European seabass was evaluated focusing on health condition after a feeding period of 34 and 62 days. Thereafter, the immune response to an inflammatory insult was also evaluated after 62 days of feeding.

In a first approach and aiming to assess the outcome of the dietary inclusion of *S. ramosissima* on the European seabass immune status, few changes were observed among dietary treatments following 34 and 62 days of feeding. In fact, dietary *S. ramosissima* could only induce an upregulation of the expression of two leucocyte receptors, *mcmf1r1* and *cd8β*, after 62 days of feeding, particularly in fish fed the highest inclusion level (ST10). Macrophage colony-stimulating factor 1 receptor 1 (*mcsf1r1*) encodes a membrane receptor for colony stimulating factor 1, a cytokine involved in the immune response of macrophages (28), whereas CD8β is a class I MHC-specific co-receptor for the T cell receptor (TCR) found in circulating T cells (29). In addition, no significant alterations were observed among the dietary treatments in other parameters measured, such as haematological profile, peripheral leucocyte numbers, or plasma humoral parameters. Taking the previous results into account, it is important to mention that the capacity of the dietary incorporation of halophytes to modulate fish immune response may be dependent on the presence of an immune stimulus such as an antigen, which was absent during the feeding period. Therefore, the authors trust that *S. ramosissima* may succeed as an alternative novel ingredient for European seabass feed, despite the few observed alterations, at least from an immune system standpoint. Moreover, data on liver oxidative stress from the present trial point to an improvement in antioxidant activity, particularly through the GSH-related defence subsystem, as described by Marçal et al. (25). Such results are supported by the antioxidant profile of *S. ramosissima* due to its phenolic and flavonoid compounds, which are essential for plant resistance to exogenous factors and endogenous pressure (30). This, together with the good immune condition of fish fed diets that include *S. ramosissima* and the similar growth performance of fish fed different dietary treatments (average final weight 43.12 ± 3.19 g), denotes an opportunity for the inclusion of *S. ramosissima* in the diets of European seabass juveniles (up to 10% of the feed). This also contributes to the concepts of a circular economy and zero waste, a fact that should be explored in further studies.

Aside from the lack of dietary effects, it may be important to discuss the observed decrease in some of the parameters measured regarding feeding time (i.e., from 34 to 62 days). For instance, the mRNA expression levels of important immune-related genes such as *il1β*, *il10*, *sod*, and *odc* decreased between sampling points, a fact possibly linked to fish growth and development over the feeding trial. These genes encode both pro- and anti-inflammatory effectors of the immune system. Interleukin (IL) 1 β and IL 10 are respectively involved in the regulation of the pro- (31) and anti-inflammatory response (32), together with the enzyme superoxide dismutase (SOD), which is implicated in the antioxidant defence against harmful superoxide (O_2_^-^) (33), representing a key mechanism involved in the regulation of the immune response. Additionally, ornithine decarboxylase (ODC), which is responsible for the decarboxylation of ornithine to putrescine in the polyamine pathway, involved in the cell proliferation and differentiation mechanisms and key during the immune response (34), was downregulated with feeding time. Such events were accompanied by a significant decrease in plasma peroxidase, a myeloperoxidase expressed mainly in neutrophils and implicated in microbial killing by these phagocytic cells (35). The authors believe that the decrease in such inflammatory indicators with time may be linked to fish growth over the 62 days as most differences were found irrespective of the dietary treatment. A similar effect was previously observed in European seabass by Machado et al. (36), in which fish fed a control diet (comparable to the one used in the present study) presented a decrease in *il1β* and *sat1* transcripts from 2 to 4 weeks of feeding. Spermine/spermidine N (1)–acetyltransferase (SAT1) is a rate-limiting enzyme involved in the regulation of the intracellular concentration of polyamines, and therefore, this could also be linked to the decrease in ODC observed in the present study. It is important to highlight the high standard deviation inherent in the results presented here that ultimately could limit the number of significant alterations observed. Nonetheless, the authors also believe that the observed variability is often inherent in tests using animals originating from commercial sources.

As previously discussed, the dietary inclusion of *S. ramosissima* could also present the added value of offering fish specific phytochemicals with immune-stimulating properties, such as hydroxycinnamic acids, phenolic acids, phenols, and flavonoids (19). With this in mind, fish were submitted to an inflammatory challenge with heat-inactivated *Phdp* after 62 days of feeding with the aim of uncovering the immunomodulatory properties of *S. ramosissima*. A clear response to the inflammatory insult was then observed in all dietary treatments. There was a general decrease in several haematological indicators and plasma peroxidase activity. This was accompanied, in all dietary treatments, by a rapid increase in the numbers of leucocytes found in the peritoneal cavity at 4 and 24 h in response to the phlogistic agent, followed by a decrease. Additionally, in response to inflammation, there was an increase in *tlr9* mRNA expression levels in response to inflammation. *Tlr9* encodes a toll-like receptor responsible for the recognition of unmethylated CpG dinucleotides from viral and bacterial DNA (37) and its downstream signal transducer, the myeloid differentiation primary response protein (38) *myd88*. Similar dynamics were also observed in the innate immune response of rock bream (*Oplegnathus fasciatus*) against rock bream iridovirus (39). In the present study, the expression of *mmp9*-coded enzyme involved in the degradation of the extracellular matrix in processes such as cell migration (40), as well as the plasma bactericidal activity, were also increased, thus being consistent with the observed increase in peritoneal leucocytes following inflammation. A similar boosted immune response in response to *Phdp* was also observed previously in seabass by Machado et al. (36).

More importantly, the dietary inclusion of *S. ramosissima* during a 62-day period led to a significant modulation of the inflammatory mechanisms. A clear improvement in the numbers of leucocytes found in the peritoneal cavity was observed, 4 h after injection, in fish fed ST10. These data were consistent with the higher *mcsf1r1* mRNA expression level at 72 h following bacterial injection. This gene encodes a receptor for colony stimulating factor I, a cytokine that controls macrophage production, differentiation, and function (28), and therefore points to an improvement in leucocyte responses to the inflammatory stimulus synergistically powered by the dietary inclusion of *S. ramosissima* dietary at 10%. Additionally, this improved inflammatory response associated with the dietary inclusion of *S. ramosissima* is further supported by the upregulation of several pro-inflammatory signals, such as the pro-inflammatory cytokine *il1β* (41) in seabass fed at all levels of *S. ramosissima* inclusion, as well as the increased expression level of the polyamine-related enzyme ODC in fish fed ST5, both at 4 h after bacterial injection. Moreover, European seabass fed both ST5 and ST10 exhibited increased expression of the antioxidant enzyme glutathione peroxidase at 72 h, and the highest dietary *Salicornia* inclusion (ST10) resulted in an upregulation of the signal transducer myeloid differentiation primary response protein (MYD88) (38), irrespective of time.

Taking into account the assumption that the dietary incorporation of *S. ramosissima* could represent an immune-related advantage for European seabass, the present study suggests that this outcome would not be possible in a non-stimulating condition. However, the authors see the value of such results as the inclusion up to 10% of the feed of the non-food fraction of *Salicornina ramosissima* collected from Praia da Areia Branca (Bunheiro-Murtosa, Portugal) did not compromise the immune status of the fish after a feeding period of 62 days and believe that such a response may be dependent on the presence of an obvious immune stimuli. In fact, when fish were challenged with inactivated bacteria and the inflammatory mechanisms were monitored, a clear improvement in the leucocyte response accompanied by an upregulation of key immune-related genes was noticed. Nonetheless, the study would benefit from a challenge study to fully evaluate the ability of the dietary inclusion of *S. ramosissima* to improve disease resistance.

Finally, it is worth mentioning that the observed results may be influenced by an inherent variability represented by the high standard deviation that ultimately could limit the number of significant observations. With that, the authors believe that the work would eventually benefit from trial repetition. Nevertheless, the authors trust the very conservative statistical test used (Tukey *post-hoc*) for data interpretation and the impact of the present study.

Considering all, the authors denote the opportunity for a practical *S. ramosissima* dietary inclusion for European seabass juveniles (up to 10% of feed) and its ability to improve its inflammatory response. Such results point to the dietary inclusion of *S. ramosissima* working in an animal production scenario, as a functional diet in immune-challenging scenarios. Moreover, as the authors believe that the observed immunomodulation could be the result of the presence of at least one bioactive compound with immune-stimulating properties, studies should be performed on the matter. Furthermore, it should also be emphasized that the observed results could be influenced not only by the origin of *S. ramosissima* but by the environmental conditions present in its growth environment.

## Data availability statement

The original contributions presented in the study are included in the article/supplementary materials, further inquiries can be directed to the corresponding author/s.

## Ethics statement

The experiments were approved by the CIIMAR Animal Welfare Committee and DGAV (ORBEA-CIIMAR_26_2018) and were carried out under license number, 0421/000/000/2020 in a registered facility (N16091.UDER). Experiments were directed by trained scientists (following FELASA category C recommendations) and were conducted according to the guidelines on the protection of animals used for scientific purposes from the European Directive, 2010/63/UE. The study was conducted in accordance with the local legislation and institutional requirements.

## Author contributions

MM: Writing – original draft. FC: Investigation, Writing – review & editing. AC: Investigation, Writing – review & editing. LR-P: Investigation, Writing – review & editing. AL: Investigation, Writing – review & editing. MP: Conceptualization, Methodology, Writing – review & editing. RR: Conceptualization, Funding acquisition, Methodology, Project administration, Writing – review & editing. BC: Conceptualization, Funding acquisition, Methodology, Resources, Writing – review & editing.
